# Reduced fucosylation in the distal intestinal epithelium of mice subjected to chronic social defeat stress

**DOI:** 10.1038/s41598-018-31403-8

**Published:** 2018-09-04

**Authors:** Yasuhiro Omata, Reiji Aoki, Ayako Aoki-Yoshida, Keiko Hiemori, Atsushi Toyoda, Hiroaki Tateno, Chise Suzuki, Yoshiharu Takayama

**Affiliations:** 1 National Institute of Livestock and Grassland Science, National Agriculture and Food Research Organization, Tsukuba, Ibaraki 305-0901 Japan; 2 Graduate School of Agricultural and Life Sciences, The University of Tokyo, Bunkyo-ku, Tokyo 113-8657 Japan; 3 Biotechnology Research Institute for Drug Discovery, National Institute of Advanced Industrial Science and Technology, Tsukuba, Ibaraki 305-8568 Japan; 4 College of Agriculture, Ibaraki University, Ami, Ibaraki 300-0393 Japan

## Abstract

Psychological stress can cause dysfunction of the gastrointestinal tract by regulating its interaction with central nervous system (brain-gut axis). Chronic social defeat stress (CSDS) is widely used to produce a rodent model of stress-induced human mood disorders and depression. We previously showed that CSDS significantly affects the intestinal ecosystem including cecal and fecal microbiota, intestinal gene expression profiles and cecal metabolite profiles. Here, we investigated whether the glycosylation pattern in the intestinal epithelium was affected in C57BL/6 mice exposed to CSDS (hereinafter referred to as CSDS mice). A lectin microarray analysis revealed that CSDS significantly reduced the reactivity of fucose-specific lectins (rAOL, TJA-II, rAAL, rGC2, AOL, AAL, rPAIIL and rRSIIL) with distal intestinal mucosa, but not with mucosa from proximal intestine and colon. Flow cytometric analysis confirmed the reduced TJA-II reactivity with intestinal epithelial cells in CSDS mice. In addition, distal intestine expression levels of the genes encoding fucosyltransferase 1 and 2 (*Fut1* and *Fut*2) were downregulated in CSDS mice. These findings suggest that CSDS alters the fucosylation pattern in the distal intestinal epithelium, which could be used as a sensitive marker for CSDS exposure.

## Introduction

Psychological stress causes gastrointestinal dysfunction, including impairment of the intestinal barrier and perturbations of the neuroendocrine system and the intestinal immune system^1–3^. The functional correlation of the central nervous system and gastrointestinal tract is called the brain-gut interaction^2,3^.

Chronic social defeat stress (CSDS) is a frequently used rodent model that mimics stress-induced human mood disorders and depression^4–7^. We previously demonstrated that CSDS has a significant effect on the population of cecal and fecal microbiota and on cecal metabolites^8^. These changes were accompanied by the suppression of genes involved in immune responses in the terminal ileum (distal small intestine)^8^. The gut occupies the largest area of contact with the outside environment, and is the largest organ of the immune system. Therefore, downregulation of genes involved in immune responses increases the vulnerability of the intestine to bacterial infection and the subsequent alteration of gut microbiota.

The intestinal epithelium is covered by a thin layer that consists of host-derived glycans and a secreted mucus layer. In a previous study using microarray analysis, we reported that the expression of *Fut2*, a gene encoding fucosyltransferase 2 (FUT2), was significantly decreased in the terminal ileum of mice subjected to CSDS^8^. FUT2 adds terminal α1,2-fucose residues to glycan chains and is responsible for most of the fucosylation in the mouse and human gut^9,10^.

In this study, we performed lectin microarray analysis to comprehensively determine the glycation pattern in the intestine and colon epithelia of C57BL/6 mice subjected to CSDS (hereinafter referred to as CSDS mice). Lectin microarray analysis is a novel technology that can be used to analyse glycans and glycoproteins through incubation with a panel of immobilised lectins that recognise specific glycan epitopes^11,12^. For example, lectin microarray analysis revealed that cell surface glycosylation in induced pluripotent stem cells was different to that in human somatic cells^13^. Here, we found that the reactivity of distal intestinal mucosa with fucose-specific lectins was diminished in CSDS mice. To confirm the results of our lectin microarray analysis, we performed flow cytometry and found that the CSDS mice displayed reduced reactivity with TJA-II, which specifically binds Fucα1-2Galβ1-4GlcNAc (H type 2).

## Results

### Development of anxiety-like behaviour induced by CSDS

**Figure 1:**
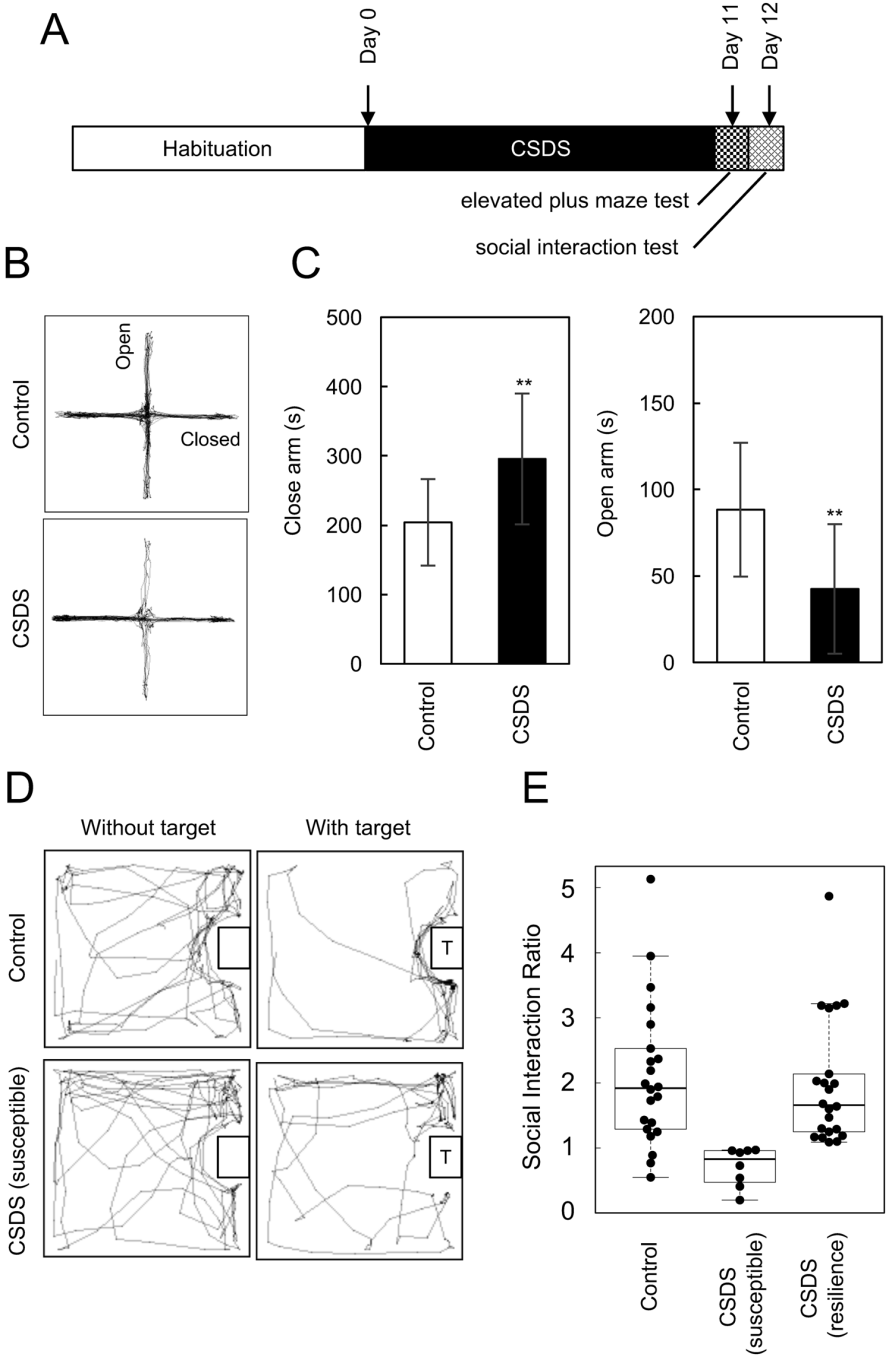
Effects of chronic social defeat stress (CSDS) on the social interaction ratio and anxiety-like behaviour in C57BL/6J mice. (**A**) Experimental design for the CSDS paradigm. (**B**) The elevated plus maze test was used to evaluate anxiety-like behaviour. Representative motion tracking data from a control and a stressed mouse (each 10 min). (**C**) Time spent in the closed and open arms of the elevated plus maze. Data are the mean ± SD of control (n = 22) and stressed (n = 30) mice. ^**^p < 0.01 vs. control. (**D**) Representative behaviour tracking images from the social interaction test in the presence (with target, T) or absence (without target) of an ICR aggressor mouse. (**E**) Social interaction ratio of defeated and control mice calculated as time spent in the interaction zone with the ICR aggressor mouse present divided by time spent in the interaction zone with the ICR aggressor mouse absent. Boxes, interquartile ranges; horizontal lines, medians; whiskers, SD.

To assess the effects of CSDS on the development of anxiety-like behaviour, we tested control and defeated C57BL/6J mice on the elevated plus maze task. As shown in Fig. 1B,C, C57BL/6J mice exposed to CSDS (n = 30) spent less time in the open arms and more time in the closed arms than control C57BL/6J mice (n = 22), indicating the development of anxiety-like behaviour in CSDS mice. The social interaction test takes advantage of the natural tendency of mice to interact with unfamiliar mice. It is well known that C57BL/6J mice subjected to CSDS can be segregated into susceptible and unsusceptible (resilient) subpopulations by the social interaction test^4,14^. Susceptible mice are defined as having a social interaction ratio less than 1. As shown in Fig. 1E, eight mice were identified as susceptible, whereas 22 mice were unsusceptible (resilient); they displayed normal social interaction behaviour (interaction ratio >1) as unstressed mice. The frequency of resilient mice was higher (22 of 30) than that in previous reports, which indicate that approximately 30% of mice are resilient to CSDS^6,8^. This difference in results may be due to the more limited exposure (5 min) of C57BL/6J mice to the ICR aggressor mice to avoid excessive injury in the present study.

### Reduced reactivity of distal intestinal mucosa with fucose-specific lectins

**Figure 2:**
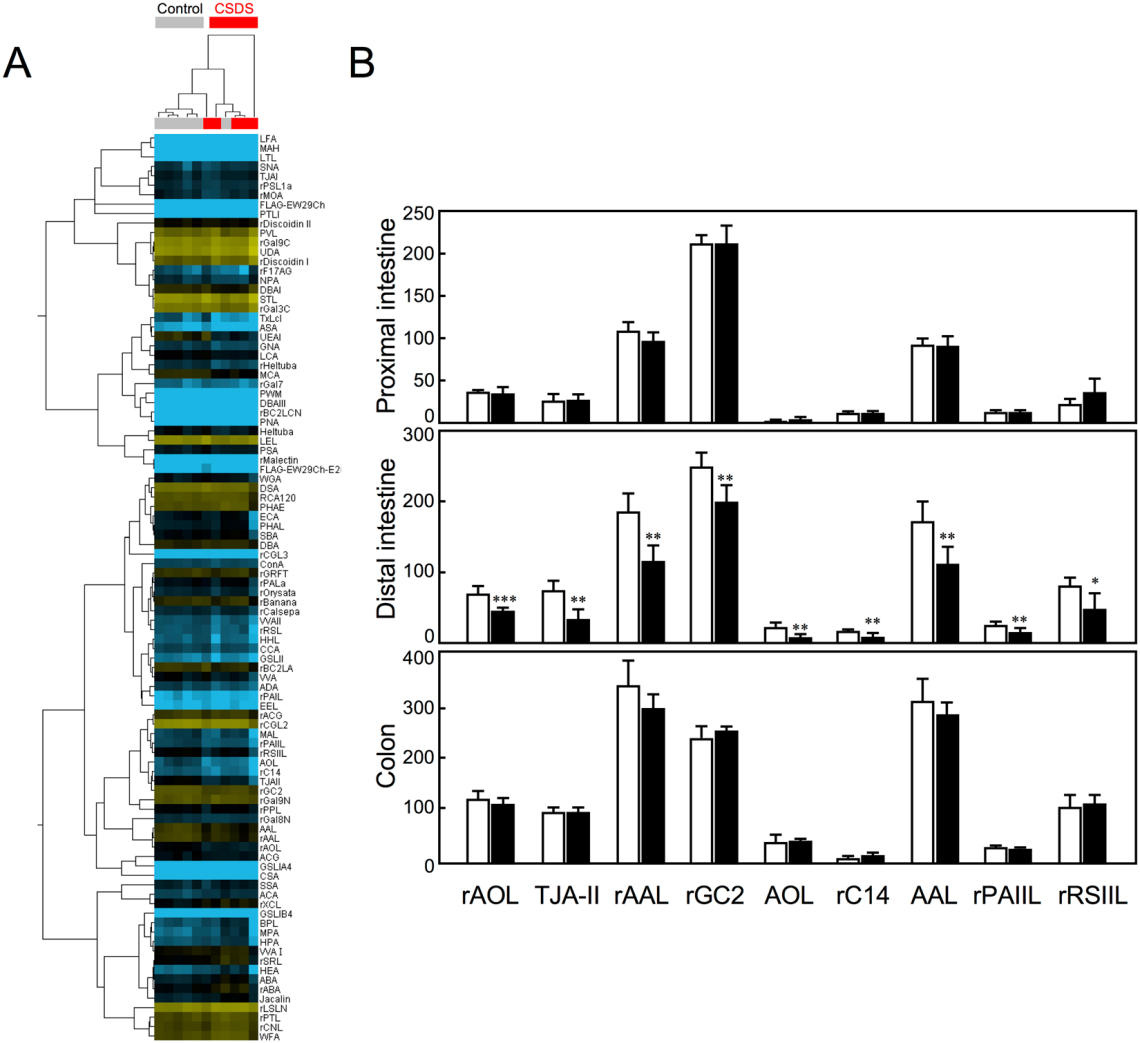
Lectin microarray analysis of CSDS mice. (**A**) Signal intensities of normalised lectin microarray data from the distal intestinal mucosa were subjected to two-dimensional hierarchical clustering and heat map analyses. The colour of each square indicates lectin-binding intensity (yellow, high; black, medium; blue, low). The 11 samples obtained from control and CSDS mice are listed in columns; the 96 lectins, in rows. (**B**) The most significantly decreased lectins were found in the distal intestine of CSDS mice. The reactivities of nine lectins (rAOL, TJA-II, rAAL, rGC2, AOL, rC14, AAL, rPAIIL and rRSIIL) with distal intestinal mucosa were compared in control and CSDS mice (middle panel). Those of the proximal intestine and colon are shown in the upper and lower panel, respectively. The intensity of the lectin signal is based on lectin array data. ^*^p < 0.05, ^**^p < 0.01, ^***^p < 0.001, vs. the control.

To determine whether our CSDS paradigm affected the glycosylation pattern in the intestinal epithelium, we extracted glycoproteins from mucosa isolated from proximal and distal intestine and colon of control mice (n = 6) and CSDS mice (n = 5) and analysed by lectin microarray analysis. The behavioural data from the individual mice used in the analysis are summarised in Supplementary Table 1. The signal intensities of the lectin microarray data were subjected to two-dimensional hierarchical clustering and heat map analyses. In distal intestine, hierarchical clustering separated the control and CSDS mice into three clusters (Fig. 2A).

The mean normalised data were processed by Student’s *t*-test to select significantly different lectins having distinct reactivities with distal intestinal mucosa from control and CSDS mice (Supplementary Table 2). As a result, nine lectins (rAOL, TJA-II, rAAL, rGC2, AOL, rC14, AAL, rPAIIL and rRSIIL) showed *p*-values less than 0.05. They showed lower signal intensities in CSDS mice than in control mice (Fig. 2B, middle panel). Among these nine lectins, eight were fucose-binding lectins. The reactivity of these lectins with mucosa from proximal intestine and colon was not significantly different between control and CSDS mice (Fig. 2B, upper and lower panels). Consistent results were obtained from independent experiment using six control and six CSDS mice (Supplemental Fig. S1). One of the lectins, TJA-II, is highly specific for Fucα1-2Galβ1-4GlcNAc (H type 2), suggesting that CSDS mice expressed reduced Fucα1-2Galβ1-4GlcNAc in distal intestinal mucosa. The reduction of the fucose signal was significantly associated with the development of anxiety-like behaviour observed in the elevated plus maze test (Fig. 2A, Supplementary Table 1). Although a difference in fucosylation of intestinal mucosa was observed between CSDS-resilient and control mice, no significant behavioural difference was observed between these two groups of mice in the social interaction test (Figs 1E, 2A, Supplementary Table 1).

### Reduced reactivity of IECs with TJA-II lectin

**Figure 3:**
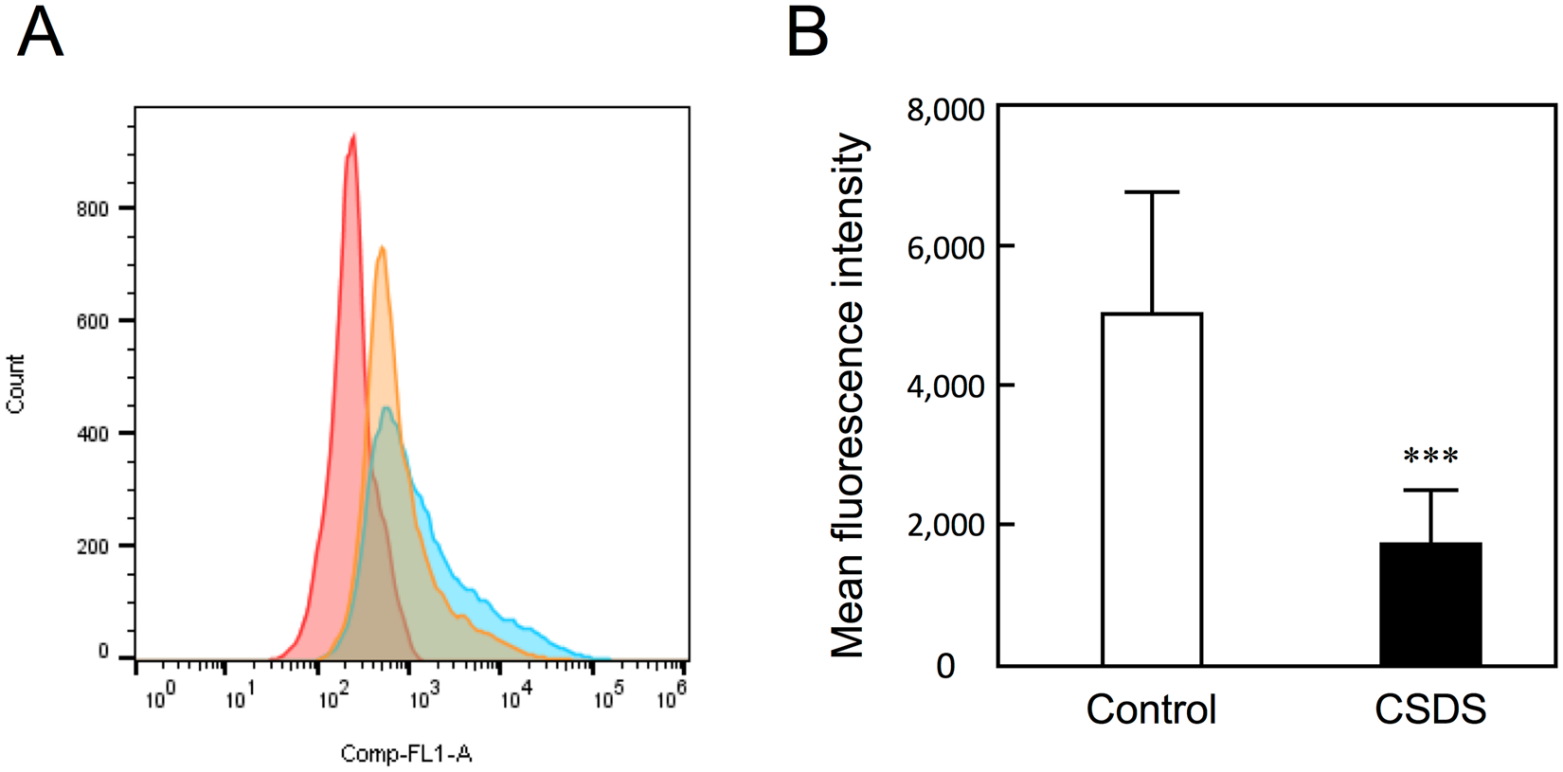
Reduced reactivity with TJA-II in the proximal intestinal epithelium of CSDS mice. (**A**) A representative flow cytometry histogram. Intestinal epithelial cells (IECs; 5 × 10^5^) isolated from control mice (n = 10; blue) and CSDS mice (n = 10; orange) were reacted with FITC-labelled lectin (TJA-II) and analysed by flow cytometry. Unstained cells are red. (**B**) Quantified mean fluorescence intensity. Data are shown as mean ± SD. Statistical significance was estimated by Student’s *t*-test. Bar indicates mean ± SD. ^***^p < 0.001 vs. the control.

With the use of flow cytometry, we quantified the binding of TJA-II lectin in distal intestinal epithelial cells (IECs) derived from CSDS mice. A histogram of the results of FITC-labelled TJA-II showed that a portion of the IECs derived from CSDS mice (n = 10) overlapped with the IECs derived from control mice (n = 10), but a negative shift in fluorescent intensity was observed in the CSDS mice (Fig. 3A). Quantified mean fluorescence intensity data showed that the average TJA-II reactivity of IECs obtained from CSDS mice was less than 40% of that detected in IECs obtained from control mice (Fig. 3B). The behavioural data from the individual mice used in flow cytometric analysis are summarised in Supplementary Table 3.

### Fucosyltransferase gene expression

**Figure 4:**
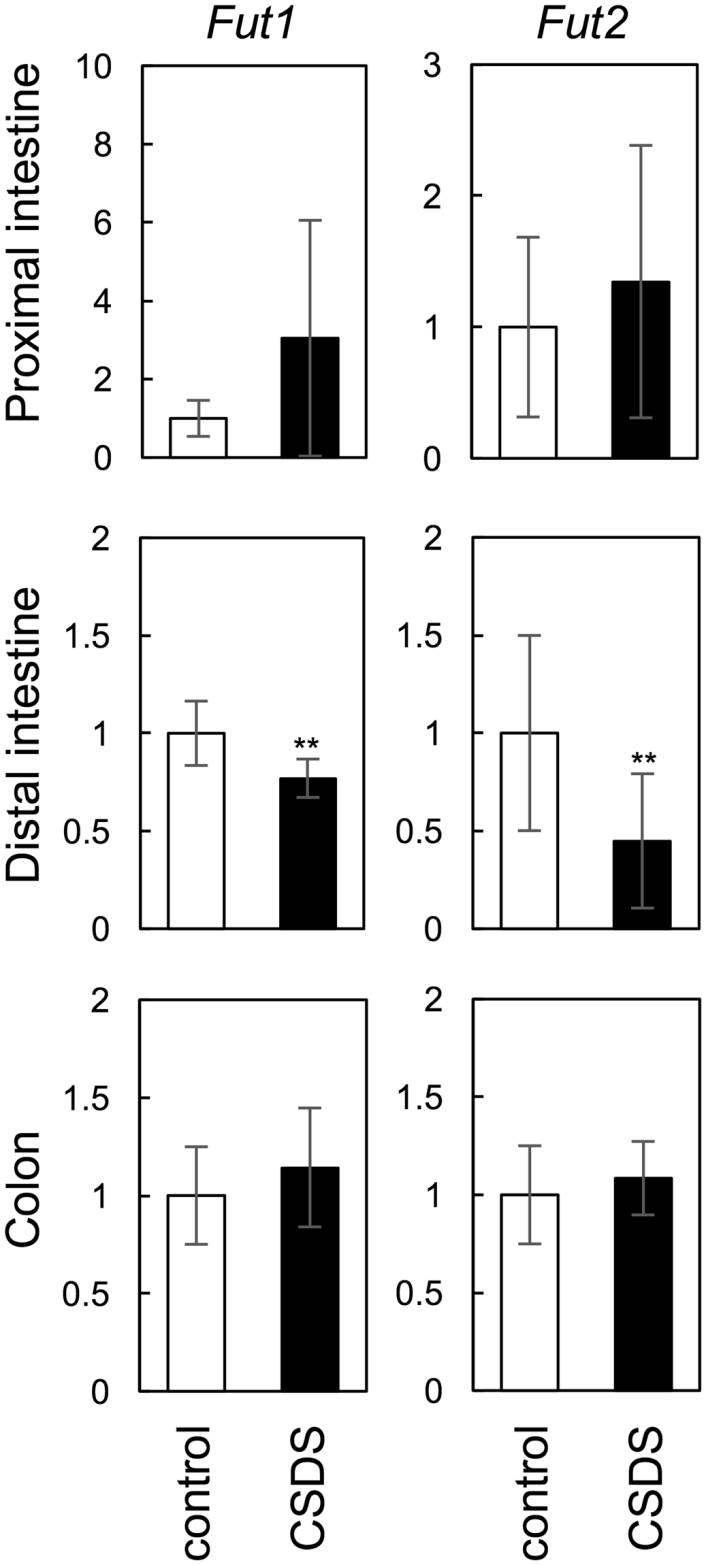
Effect of CSDS on fucosyltransferase gene expression in the proximal and distal intestine and colon. The relative mRNA levels of fucosyltransferases *Fut1* and *Fut2* were determined by qPCR after normalisation to *Gapdh* mRNA levels. All values are means ± SD (n = 10 per group); ^**^p < 0.01 vs. the control.

We performed quantitative polymerase chain reaction (qPCR) analysis to investigate the effect of CSDS on the mRNA expression of fucosyltransferases in the proximal and distal intestine and colon. CSDS attenuated *Fut1* and *Fut2* mRNA expression levels in the distal intestine but not in the proximal intestine or colon (Fig. 4). These observations agreed with the results of our lectin microarray analysis (Fig. 2) and suggested that reduced fucosylation in the distal intestinal epithelium of CSDS mice resulted from suppression of fucosyltransferase gene expression. The behavioural data from the individual mice used in qPCR analysis are summarised in Supplementary Table 3.

## Discussion

Psychological stress, including CSDS, affects the diverse physiological functions of the gastrointestinal tract^1^. Here, we found that CSDS decreased the reactivity of intestinal mucosa with fucose-specific lectins, especially with α1,2-linked lectins (Fig. 2B). The α1,2-linked fucosylation is a major glycosylation detected on the surface of IECs and secreted proteins^10^. The addition of an α1,2-linked fucose to terminal galactose residues of glycan is catalysed by two α1,2-fucosyltransferases (FUT1 and FUT2). We found that the effect of CSDS on fucosylation was associated with the downregulation of both *Fut1* and *Fut2* in the distal intestine (Fig. 4). These observations suggested that CSDS affected the fucosylation pattern in the IECs of the distal intestine by regulating fucosyltransferase expression.

Intestinal fucosylation is regulated by many intestinal environmental stresses, including tissue injury, bacterial infection and inflammation^9,10^. FUT1 mediates the α1,2-linked fucosylation in M cells^15^. Therefore, FUT2 is considered the enzyme primarily responsible for fucosylation in IECs. Thus, FUT1/2 plays an important role in maintaining intestinal homeostasis by regulating the fucosylation level in response to alterations of intestinal environment.

Cross-talk between IECs and mucosal immune cells plays an important role in the maintenance of intestinal homeostasis. The induction of α1,2-fucose in IECs was regulated by a subset of mucosal immune cells, especially group 3 innate lymphoid cells (ILC3)^10,16^, implying that α1,2-fucose in IECs act as a biological and interactive molecule for the host–microbe cross-communication.

We demonstrated here that a CSDS-induced reduction in reactivity with fucose-specific lectins was significantly associated with the development of anxiety-like behaviour observed in the elevated plus maze test (Supplementary Table 1, Fig. 2B), whereas there was no significant difference in the reactivity of fucose-specific lectins between susceptible and resilient mice as determined by a social interaction test (Supplementary Table 1, Fig. 2B). We speculate that α1,2-linked fucosylation of the distal intestinal IECs could be a sensitive marker of CSDS exposure. Reduction of α1,2-linked fucosylated glycoproteins in distal intestine may affect distribution of microbiota in the lower distal intestine, which may be involved in the development of CSDS-induced anxiety.

The *Fut2* has been identified as a risk factor in Crohn’s disease, a chronic gastrointestinal inflammatory disease that is characterised by abnormal function of the immune system^17,18^. As described above, mucosal immune cells have the ability to regulate *Fut2* expression and subsequent α1,2-linked fucosylation of IECs by interleukin 22–dependent mechanisms^16^. These findings suggest that the levels of α1,2-linked fucosylation in IECs are correlated with the activity of the host’s innate immune system. We previously showed that our CSDS paradigm results in the suppression of genes involved in immune responses in the terminal ileum^8^. The results of the present study suggest that intestinal fucosylation is regulated by cross talk between the host intestinal immune system in CSDS mice.

In contrast to the distal intestine, no effect of CSDS on reactivity with fucose-specific lectins or mRNA expression of fucosyltransferases (*Fut1* and *Fut2*) was detected in the colon or proximal intestine (Fig. 2B). This observation agrees with the finding that α1,2-linked fucose expression is predominantly observed in the ileum (distal intestine) rather than in the duodenum or jejunum^16^, and suggests that the expression of *Fut1* and *Fut2* alters the microbiota population along with the downward flow of intestinal contents.

Post-translational modifications of protein play an important role in diverse physiological functions by regulating protein-protein, protein-macromolecule and host-microbiota interactions. Fucosylation is a fundamental post-translational modification. However, the role of protein fucosylation in the onset of behavioural or emotional disorders is poorly understood. Mice with a *Fut8* (α1,6-fucosyltransferase) deficiency exhibit multiple behavioural abnormalities, including decreased social interaction, suggesting that α1,6-linked fucosylation is significantly involved in behavioural responses mediated by the central nervous system^19^. The present study is the first to show the correlation of intestinal α1,2-fucosylation levels with behavioural abnormalities.

## Methods

All animal procedures were conducted in accordance with the animal experimentation guidelines of the National Agriculture and Food Research Organization (Ibaraki, Japan). The protocol was approved by the Animal Care Committee, National Institute of Livestock and Grassland Science (Ibaraki, Japan; permit number:1711D017).

### Chronic social defeat stress

Male C57BL/6J mice (7 weeks of age) and male Slc:ICR (ICR) mice (older than 5 months of age) were purchased from SLC Japan (Shizuoka, Japan). An experimental C57BL/6J intruder mouse was exposed to an ICR aggressor mouse for 5 min^4^. After the 5-min-defeat session, the ICR mouse and the intruder C57BL/6J mouse were kept in the same cage but were separated by a divider for the remainder of the day. This procedure was repeated for 10 consecutive days, using a different aggressor ICR mouse every day (Fig. 1A).

### Social interaction test

The social interaction test was performed the day after the completion of CSDS as previously described^6^. The social interaction ratio was calculated as follows: duration of time spent in the interaction zone with the ICR mouse present divided by the duration of time spent in the interaction zone with the ICR mouse absent.

### Elevated plus maze test

The elevated plus maze test was performed as previously described on the day after the social interaction test^6^. The time spent in and the number of explorations of the open and closed arms was determined by video-tracking software (O’Hara & Co., Ltd., Tokyo, Japan).

### Tissue sampling

Animals were euthanised by cervical dislocation under isoflurane anaesthesia. Six-centimetre-long sections of proximal and distal small intestine and colon were dissected. The proximal 1 cm segments were immediately immersed in RNAlater (Qiagen, Valencia, CA, USA), and the remaining 5 cm of each tissue was used for IEC collection.

### Preparation of intestinal mucosa

A portion of the 5 cm section of intestine (proximal and distal small intestine, and colon) was longitudinally opened. The intestinal mucosa was scraped from the substratum using a cover glass and suspended in cold phosphate-buffered saline (PBS). The supernatant was removed after centrifugation (200 × *g*, 5 min at 4 °C), and cells were immediately frozen in liquid nitrogen and kept at −80 °C until used in assays.

### Lectin microarray production and analysis

The lectin microarray was produced as described previously^11,13^. Hydrophobic fractions from intestinal mucosa were prepared, labelled with Cy3 and applied to the lectin microarray as previously described^14^. Fluorescence images were acquired using an evanescent field-activated fluorescence scanner (Bio-REX Scan 200; Rexxam, Osaka, Japan).

### Flow cytometric analysis

The IECs were dissociated by a mechanical procedure according to a protocol described by Roulis *et al*.^20^. The proximal intestine (5 cm) was removed intact, flushed with calcium- and magnesium-free Hanks’ balanced salt solution (HBSS; Life Technologies, Grand Island, NY, USA) containing 2% fetal bovine serum (FBS), opened longitudinally and cut into pieces 0.5 cm in length. The tissue was washed and incubated in HBSS containing 2% FBS, 0.5 mM EDTA and 1 mM dithiothreitol with shaking at 37 °C for 45 min. After vigorous shaking, the detached cells were separated from intestinal fragments by gauze filtration. The cell suspension was centrifuged at 1,800 × *g* for 10 min. The pellet was resuspended in 4 mL of 25% Percoll (GE Healthcare) and layered on 2 mL of 40% Percoll. After centrifugation (600 × *g* for 10 min), IECs were collected from the interphase. The reactivity of IECs with TJA-II lectin was evaluated by flow cytometry (Gallios, Beckman Coulter, Miami, FL, USA) and analysed using FlowJo software (Tree Star, Ashland, OR, USA). A suspension of IECs (5 × 10^5^ cells) was incubated at 4 °C for 1 hr with 10 µg/mL FITC-labelled TJA-II lectin in 50 µL of PBS containing 1% bovine serum albumin.

### Quantitative real-time PCR

The mRNA levels of fucosyltransferases were measured by quantitative real-time PCR (qPCR). Total RNA was isolated from whole proximal and distal intestine and colon. Total RNA was extracted with an RNeasy Mini Kit (Qiagen) by following the manufacturer’s instructions. The cDNA was synthesised from total RNA (250 ng) using ReverTra Ace qPCR RT Master Mix (Toyobo, Osaka, Japan), and qPCR was performed using Thunderbird SYBR qPCR Mix (Toyobo) and a CFX96 real-time PCR detection system (Bio-Rad, Hercules, CA, USA). The cycling program used was one cycle at 95 °C for 60 s, followed by 40 cycles at 95 °C for 15 s and 60 °C for 50 s. The dissociation curve was obtained by heating amplicon 65 to 95 °C at every 0.5 °C increase for 5 s. The relative amount of each transcript was normalised to the amount of *Gapdh* in the same cDNA. The primer sequences were as follows: *Fut1*, 5′-TCAACTTGCTGGAAATGCTG-3′ (forward) and 5′-GAGTCGGAAGAGACTGTGGC-3′ (reverse); *Fut2*, 5′-GAGTCAAGGGGAGGGAGAAC-3′ (forward) and 5′-AACTTGGTGAGGGGACTGTG-3′ (reverse); *Gapdh*, 5′-AGGTCGGTGTGAACGGATTTG-3′ (forward) and 5′-GGGGTCGTTGATGGCAACA-3′ (reverse).

### Statistical analysis

Statistical analyses were performed with unpaired, two-tailed Student’s *t-*tests. Q-values were calculated using Benjamini-Hochberg false discovery rate (FDR) procedure.

## Electronic supplementary material

Dataset 1

## Data Availability

The datasets analysed during the current study are available from the corresponding author on reasonable request.

## References

[1] Mayer EA (2011). Gut feelings: the emerging biology of gut-brain communication. Nat. Rev. Neurosci..

[2] Moloney RD, Desbonnet L, Clarke G, Dinan TG, Cryan JF (2014). The microbiome: stress, health and disease. Mamm. Genome..

[3] Mayer EA, Tillisch K, Gupta A (2015). Gut/brain axis and the microbiota. J. Clin. Invest..

[4] Golden SA, Covington HE, Berton O, Russo SJ (2011). A standardized protocol for repeated social defeat stress in mice. Nat. Protoc..

[5] Chaouloff F (2013). Social stress models in depression research: what do they tell us?. Cell Tissue Res..

[6] Goto T (2014). Subchronic and mild social defeat stress accelerates food intake and body weight gain with polydipsia-like features in mice. Behav. Brain Res..

[7] Toyoda A (2017). Social defeat models in animal science: What we have learned from rodent models. Anim. Sci. J..

[8] Aoki-Yoshida A (2016). Omics studies of the murine intestinal ecosystem exposed to subchronic and mild social defeat stress. J. Proteome Res..

[9] Pickard JM, Chervonsky AV (2015). Intestinal fucose as a mediator of host-microbe symbiosis. J. Immunol..

[10] Goto Y, Uematsu S, Kiyono H (2016). Epithelial glycosylation in gut homeostasis and inflammation. Nat. Immunol..

[11] Kuno A (2005). Evanescent-field fluorescence-assisted lectin microarray: a new strategy for glycan profiling. Nat. Methods..

[12] Hirabayashi J, Kuno A, Tateno H (2015). Development and applications of the lectin microarray. Top Curr. Chem..

[13] Tateno H (2011). Glycome diagnosis of human induced pluripotent stem cells using lectin microarray. J. Biol. Chem..

[14] Franklin TB, Saab BJ, Mansuy IM (2012). Neural mechanisms of stress resilience and vulnerability. Neuron..

[15] Terahara K (2011). Distinct fucosylation of M cells and epithelial cells by Fut1 and Fut2, respectively, in response to intestinal environmental stress. Biochem. Biophys. Res. Commun..

[16] Goto Y (2014). Innate lymphoid cells regulate intestinal epithelial cell glycosylation. Science..

[17] Tong M (2014). Reprograming of gut microbiome energy metabolism by the *FUT2* Crohn’s disease risk polymorphism. ISME J..

[18] McGovern DP (2010). International IBD Genetics Consortium. *Fucosyltransferase 2* (*FUT2*) non-secretor status is associated with Crohn’s disease. Hum. Mol. Genet..

[19] Fukuda T (2011). α1,6-fucosyltransferase-deficient mice exhibit multiple behavioral abnormalities associated with a schizophrenia-like phenotype: importance of the balance between the dopamine and serotonin systems. J. Biol. Chem..

[20] Roulis M (2011). Intestinal epithelial cells as producers but not targets of chronic TNF suffice to cause murine Crohn-like pathology. Proc. Natl. Acad. Sci. USA.

