# Supplementary material for: Reduced fucosylation in the distal intestinal epithelium of mice subjected to chronic social defeat stress
Source: Sci Rep. 2018 Sep 4;8:13199. doi: 10.1038/s41598-018-31403-8 (PMC6123462; doi:10.1038/s41598-018-31403-8)
Supplement: Supplementary file 1 — Dataset 1 [file 41598_2018_31403_MOESM1_ESM.doc]

**Reduced fucosylation in the distal intestinal epithelium of mice subjected to chronic social defeat stress**

Yasuhiro Omata, Reiji Aoki, Ayako Aoki-Yoshida, Keiko Hiemori, Atsushi Toyoda, Hiroaki Tateno, Chise Suzuki and Yoshiharu Takayama

Supplementary Table 1. Behavioural data from mice used for the lectin array analysis

Elevated plus maze test

| Mouse no. | Time spent in closed arms (s) | Time spent in open arms (s) |
| --- | --- | --- |
| no stress_18 | 196.5 | 80.5 |
| no stress_19 | 216.0 | 89.0 |
| no stress_20 | 164.5 | 136.5 |
| no stress_21 | 243.5 | 48.0 |
| no stress_22 | 165.5 | 81.5 |
| no stress_23 | 101.0 | 99.5 |
| stress_29 | 209.5 | 81.0 |
| stress_30 | 296.5 | 75.5 |
| stress_32 | 496.0 | 0 |
| stress_33 | 213.5 | 63.5 |
| stress_34 | 467.5 | 7.0 |

Social interaction test

| Mouse no. | Time spent in interaction zone (s) | Social interaction score |
| --- | --- | --- |
| no stress_18 | 87.0 | 3.95 |
| no stress_19 | 113.5 | 1.79 |
| no stress_20 | 94.0 | 1.90 |
| no stress_21 | 68.5 | 1.25 |
| no stress_22 | 64.0 | 2.37 |
| no stress_23 | 99.5 | 1.73 |
| stress_29 * | 67.0 | 3.19 |
| stress_30 | 57.5 | 0.97 |
| stress_32 * | 95.0 | 4.87 |
| stress_33 * | 59.5 | 3.22 |
| stress_34 * | 78.0 | 2.14 |

*resilient mouse.

Supplementary Table 2. Lectins used in the microarray analysis

| Lectin | *p*-value | *t*-value | Specificity |  |
| --- | --- | --- | --- | --- |
| rAOL | 0.00083757 | 4.908674857 | α1-2Fuc (H), α1-3Fuc (Lex), α1-3Fuc (Lea) |  |
| TJAII | 0.001440798 | 4.52289514 | α1-2Fuc |  |
| rAAL | 0.002231998 | 4.222255696 | α1-2Fuc (H), α1-3Fuc (Lex), α1-3Fuc (Lea) |  |
| rGC2 | 0.003900578 | 3.850961317 | α1-2Fuc (H), αGalNAc (A), αGal (B) |  |
| AOL | 0.005521606 | 3.625802259 | α1-2Fuc (H), α1-3Fuc (Lex), α1-3Fuc (Lea) |  |
| rC14 | 0.005760148 | 3.598682274 | Branched LacNAc |  |
| AAL | 0.006062879 | 3.565914765 | α1-2Fuc (H), α1-3Fuc (Lex), α1-4Fuc (Lea) |  |
| rPAIIL | 0.008787281 | 3.330843215 | αMan, α1-2Fuc (H), α1-3Fuc (Lex), α1-4Fuc (Lea) |  |
| rRSIIL | 0.010298734 | 3.231448007 | α1-2Fuc (H), α1-3Fuc (Lex), α1-3Fuc (Lea) |  |
| MAL | 0.063094536 | 2.119444139 | α2-3Sia |  |
| rGal8N | 0.063100153 | 2.119389342 | α2-3Sia |  |
| MCA | 0.066878906 | 2.083552605 | α1-2Fuc |  |
| ConA | 0.079657726 | 1.975317297 | M3, Manα1-2Manα1-3(Manα1-6)Man, GlcNAcβ1-2Manα1-3(Manα1-6)Man |  |
| rXCL | 0.083961722 | -1.94257445 | Core1,3, agalacto N-glycan |  |
| ADA | 0.115959331 | 1.739406398 | α2-6Sia, Forssman, A, B |  |
| MAH | 0.142850773 | 1.605480685 | α2-3Sia |  |
| HHL | 0.161366324 | 1.525943579 | Manα1-3Man, Manα1-7Man |  |
| rCGL2 | 0.176865209 | 1.585192731 | GalNAca1-3Gal (A), PolyLacNAc |  |
| rDiscoidin I | 0.191502086 | -1.542963481 | Gal |  |
| rHeltuba | 0.193163302 | 1.406463715 | Manα1-3Man |  |
| GSLII | 0.200346837 | 1.381858257 | GlcNAcβ1-4Man |  |
| UDA | 0.206844117 | -1.360245453 | (GlcNAc)n |  |
| PVL | 0.209161714 | -1.436576614 | Sia, GlcNAc |  |
| rPSL1a | 0.218722447 | 1.322182171 | α2-6Sia |  |
| ECA | 0.235936886 | 1.269995033 | βGal |  |
| Jacalin | 0.237777972 | -1.264603303 | Galβ1-3GalNAc (T), GalNAca (Tn) |  |
| ACG | 0.242532465 | 1.311392802 | α2-3Sia |  |
| CCA | 0.251508303 | 1.306812375 | Galactosylated N-glycans up to triantenna |  |
| STL | 0.253719928 | -1.290917554 | Polylactosamine, (GlcNAc)n |  |
| PWM | 0.256457576 | 1.211759019 | (GlcNAc)n |  |
| rLSLN | 0.257662108 | -1.208460874 | LacNAc, polylactosamine |  |
| rRSL | 0.271371004 | 1.246949592 | αMan, α1-2Fuc (H), α1-3Fuc (Lex), α1-4Fuc (Lea) |  |
| ACA | 0.275870037 | -1.16006523 | Galβ1-3GalNAc (T) |  |
| UEAI | 0.275910223 | 1.159961299 | α1-2Fuc |  |
| GNA | 0.286276188 | 1.133546613 | Manα1-3Man, Manα1-6Man |  |
| rMOA | 0.301496719 | 1.09610062 | αGal (B) |  |
| ASA | 0.30282003 | 1.092915597 | Galβ1-4GlcNAcβ1-2Man |  |
| rGal9C | 0.364685663 | -1.014314864 | PolyLacNAc, Branched LacNAc |  |
| rACG | 0.373027331 | 0.966118266 | α2-3Sia |  |
| LFA | 0.389283142 | 0.904534034 | Sia |  |
| rGRFT | 0.401658641 | 0.911038029 | Man |  |
| rMαlectin | 0.424026347 | -0.887982026 | Glcα1-2Glc |  |
| TJAI | 0.463692227 | 0.765292905 | α2-6Sia |  |
| VVAII | 0.467143445 | 0.788099954 | Man, Agalacto |  |
| FLAG-E20K | 0.480016646 | -0.775068698 | 6-sulfo-Gal |  |
| VVA | 0.499331616 | 0.703847524 | α,βGalNAc (A, Tn, LacDiNAc) |  |
| DBA | 0.499927457 | -0.702844245 | α,βGalNAc (A, Tn, LacDiNAc) |  |
| rCalsepa | 0.501887394 | 0.721414528 | Biantenna with bisecting GlcNAc |  |
| LTL | 0.509880159 | 0.68619436 | Lex, Ley |  |
| rPALa | 0.511957015 | 0.705643967 | Man5, biantenna |  |
| rBC2LA | 0.513376007 | 0.680393714 | αMan, High-man |  |
| TxLcI | 0.515001236 | 0.677705206 | Galactosylated N-glycans up to triantenna |  |
| rPPL | 0.523737128 | 0.692097473 | α,βGalNAc (A, Tn, LacDiNAc) |  |
| rCGL3 | 0.53284538 | 0.648518537 | LacDiNAc |  |
| SNA | 0.539860143 | 0.637204862 | α2-6Sia |  |
| rGal3C | 0.540747124 | -0.635780491 | LαcNAc, polylactosamine |  |
| HPA | 0.551157058 | -0.619164519 | αGalNAc (A, Tn) |  |
| RCA120 | 0.566097359 | 0.595630181 | βGal |  |
| GSLIA4 | 0.568820123 | 0.59137932 | αGalNAc (A, Tn) |  |
| LCA | 0.577775238 | 0.577477994 | α1-6Fuc up to biantenna |  |
| DSA | 0.580718571 | 0.572935074 | GlcNAcβ1-6Man (Tetraantenna) |  |
| MPA | 0.581806921 | -0.571258456 | Galβ1-3GalNAc (T), GalNAca (Tn) |  |
| LEL | 0.598146271 | -0.546289707 | Polylactosamine, (GlcNAc)n | |
| rPAIL | 0.601413005 | 0.541341823 | α,βGal, αGalNAc (Tn) | |
| GSLIB4 | 0.603035342 | 0.538889885 | αGal (B) | |
| PSA | 0.624414855 | -0.506893856 | α1-6Fuc up to biantenna | |
| rABA | 0.637243236 | -0.487963024 | Galβ1-3GalNAc (T), GlcNAc | |
| rGal9N | 0.641035069 | 0.482403783 | GalNAca1-4Gal (A), PolyLacNAc | |
| PHAE | 0.641782906 | 0.481309281 | bisecting GlcNAc | |
| CSA | 0.643383003 | 0.478969543 | Rhamnose, Galα1-4Gal | |
| PHAL | 0.648988104 | 0.470795748 | GlcNAcβ1-6Man (Tetraantenna) | |
| rGal7 | 0.650288625 | 0.468904112 | Type1 LacNAc, chondroitin polymer | |
| HEA | 0.674642552 | -0.433805438 | Galβ1-3GalNAc (T) | |
| ABA | 0.688045794 | -0.414736925 | Galβ1-3GalNAc (T), GlcNAc | |
| rOrysata | 0.706278246 | 0.400360851 | Manα1-3Man, Highman, biantenna | |
| EEL | 0.723577435 | -0.364949923 | αGal (B) | |
| SBA | 0.729133333 | 0.357257027 | α,βGalNAc (A, Tn, LacDiNAc) | |
| rSRL | 0.744316144 | -0.336350336 | Core1,3, agalacto N-glycan | |
| WGA | 0.755290128 | 0.321339562 | (GlcNAc)n, polySia | |
| WFA | 0.771024558 | -0.299954152 | Terminal GalNAc, LacDiNAc | |
| SSA | 0.781854162 | 0.28532295 | α2-6Sia | |
| PNA | 0.796458837 | -0.26569679 | Galβ1-3GalNAc (T) | |
| DBAI | 0.820620842 | -0.23947436 | High-man | |
| rCNL | 0.823750446 | -0.229315102 | α,βGalNAc (A, Tn, LacDiNAc) | |
| DBAIII | 0.825135838 | 0.227477626 | Maltose | |
| rBanana | 0.839124704 | 0.213720505 | Manα1-2Manα1-3(6)Man | |
| Heltuba | 0.856219226 | -0.186460044 | Manα1-3Man | |
| rF17AG | 0.866463175 | -0.17302154 | GlcNAc | |
| rDiscoidin II | 0.894084177 | -0.136951072 | LacNAc, Galβ1-3GalNAc (T), GalNAc (Tn) | |
| VVAⅠ | 0.914452004 | 0.110481469 | GalNAcβ1-3(4)Gal | |
| BPL | 0.918130626 | -0.105710548 | Galβ1-3GlcNAc(GalNAc), α/βGalNAc | |
| rBC2LCN | 0.967514003 | 0.041873223 | Fuc α1-2Galβ1-3GlcNAc (GalNAc) | |
| NPA | 0.985583773 | -0.018577128 | Manα1-3Man | |
| rPTL | 0.99827492 | -0.002222843 | α1-6Fuc | |

Abbreviations: Gal, D-galactose; GalNAc, N-acetyl-galactosamine; GlcNAc, N-acetyl-glucosamine; Fuc, L-fucose; Glc, D-glucose; Sia, sialic acid; LacNAc, N-acetyl-lactosamine.

Supplementary Table 3. The list of most significantly decreased 10 lectins in the distal intestine of CSDS mice.

| Lectin | *p*-value | *q*-value |
| --- | --- | --- |
| rAOL | 0.00083757 | 0.06771751 |
| TJAII | 0.001440798 | 0.06771751 |
| rAAL | 0.002231998 | 0.06993594 |
| rGC2 | 0.003900578 | 0.0814158 |
| AOL | 0.005521606 | 0.0814158 |
| rC14 | 0.005760148 | 0.0814158 |
| AAL | 0.006062879 | 0.0814158 |
| rPAIIL | 0.008787281 | 0.10325055 |
| rRSIIL | 0.010298734 | 0.10756456 |

Supplementary Table 4. Behavioural data from mice used for the FACS and qPCR analysis

Elevated plus maze test

| Mouse no. | Time spent in closed arms (s) | Time spent in open arms (s) |
| --- | --- | --- |
| no stress_77 | 181.5 | 54.0 |
| no stress_79 | 310.0 | 57.5 |
| no stress_80 | 196.5 | 107.5 |
| no stress_81 | 168.5 | 105.5 |
| no stress_82 | 238.0 | 116.0 |
| no stress_83 | 184.5 | 139.0 |
| no stress_84 | 172.5 | 152.0 |
| no stress_85 | 148.0 | 138.0 |
| no stress_86 | 326.5 | 44.0 |
| no stress_87 | 169.5 | 146.5 |
| stress_88 | 333.0 | 47.0 |
| stress_89 | 309.5 | 59.5 |
| stress_90 | 298.0 | 63.5 |
| stress_91 | 188.5 | 155.5 |
| stress_92 | 377.5 | 47.5 |
| stress_93 | 120.5 | 109.5 |
| stress_94 | 226.5 | 99.5 |
| stress_95 | 447.0 | 22.0 |
| stress_96 | 435.5 | 22.5 |
| stress_97 | 355.0 | 42.0 |

Social interaction test

| Mouse no. | Time spent in interaction zone (s) | Social interaction score |
| --- | --- | --- |
| no stress_77 | 110.5 | 1.94 |
| no stress_79 | 5.5 | 0.55 |
| no stress_80 | 80.5 | 3.16 |
| no stress_81 | 88.5 | 1.99 |
| no stress_82 | 41.5 | 0.77 |
| no stress_83 | 80.5 | 1.18 |
| no stress_84 | 109.0 | 1.43 |
| no stress_85 | 74.5 | 2.33 |
| no stress_86 | 114.0 | 2.19 |
| no stress_87 | 79.5 | 5.13 |
| stress_88 * | 122.0 | 1.10 |
| stress_89 | 110.5 | 1.99 |
| stress_90 * | 121.0 | 1.64 |
| stress_91 | 63.5 | 0.96 |
| stress_92 * | 106.5 | 1.09 |
| stress_93 * | 105.0 | 1.19 |
| stress_94 * | 118.0 | 1.90 |
| stress_95 * | 145.0 | 1.68 |
| stress_96 | 76.5 | 0.93 |
| stress_97 * | 67.0 | 3.19 |

*resilient mouse.


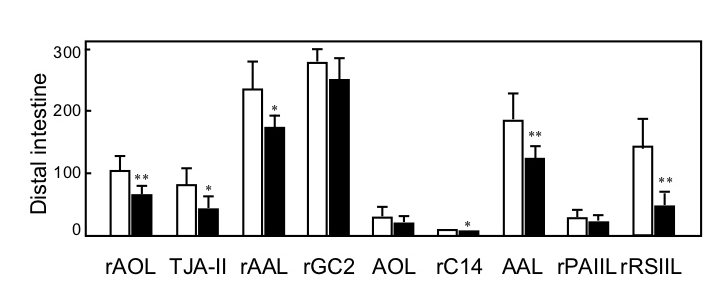
Supplemental Figure S1

The reactivities of nine lectins (rAOL, TJA-II, rAAL, rGC2, AOL, rC14, AAL, rPAIIL and rRSIIL) with distal intestinal mucosa were compared in control mice (open bar, n=6) and CSDS mice (closed bar, n=6). The intensity of the lectin signal is based on lectin array data. *p < 0.05, **p < 0.01 vs. the control.
